# Development of Polymer–Lipid Hybrid Nanoparticles for Large-Sized Plasmid DNA Transfection

**DOI:** 10.1021/acsami.3c14714

**Published:** 2023-12-23

**Authors:** Masatoshi Maeki, Shuya Uno, Kaisei Sugiura, Yusuke Sato, Yoichiro Fujioka, Akihiko Ishida, Yusuke Ohba, Hideyoshi Harashima, Manabu Tokeshi

**Affiliations:** † Division of Applied Chemistry, Faculty of Engineering, Hokkaido University, Kita 13 Nishi 8, Kita-ku, Sapporo 060-8628, Japan; ‡ JST PRESTO, 4-1-8 Honcho, Kawaguchi, Saitama 332-0012, Japan; § Institute of Materials Structure Science, High Energy Accelerator Research Organization (KEK), Tsukuba, Ibaraki 305-0801, Japan; ∥ Graduate School of Chemical Sciences and Engineering, Hokkaido University, Kita 13 Nishi 8, Kita-ku, Sapporo 060-8628, Japan; ⊥ Faculty of Pharmaceutical Sciences, Hokkaido University, Kita 12 Nishi 8, Kita-ku, Sapporo 060-0812, Japan; # Department of Cell Physiology, Faculty of Medicine and Graduate School of Medicine, Hokkaido University, Kita 15 Nishi 7, Kita-ku, Sapporo 060-8638, Japan

**Keywords:** lipid nanoparticles, polymer−lipid hybrid nanoparticle, large plasmid DNA transfection, core−shell nanoparticle, microfluidic device

## Abstract

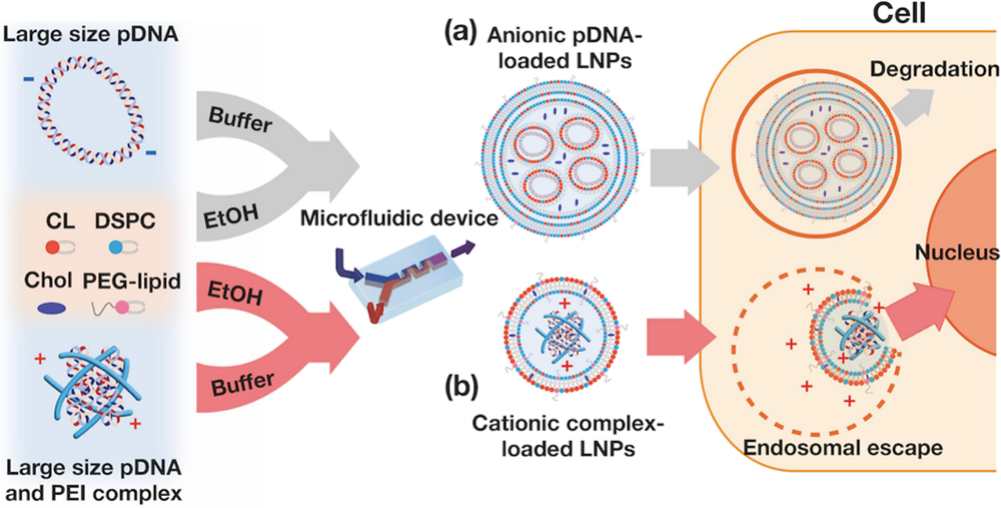

RNA and DNA delivery technologies using lipid nanoparticles (LNPs) have advanced significantly, as demonstrated by their successful application in mRNA vaccines. To date, commercially available RNA therapeutics include Onpattro, a 21 bp siRNA, and mRNA vaccines comprising 4300 nucleotides for COVID-19. However, a significant challenge remains in achieving efficient transfection, as the size of the delivered RNA and DNA increases. In contrast to RNA transfection, plasmid DNA (pDNA) transfection requires multiple steps, including cellular uptake, endosomal escape, nuclear translocation, transcription, and translation. The low transfection efficiency of large pDNA is a critical limitation in the development of artificial cells and their cellular functionalization. Here, we introduce polymer–lipid hybrid nanoparticles designed for efficient, large-sized pDNA transfection. We demonstrated that LNPs loaded with positively charged pDNA-polycation core nanoparticles exhibited a 4-fold increase in transfection efficiency for 15 kbp pDNA compared with conventional LNPs, which encapsulate a negatively charged pDNA-polycation core. Based on assessments of the size and internal structure of the polymer–lipid nanoparticles as well as hemolysis and cellular uptake analysis, we propose a strategy to enhance large-sized pDNA transfection using LNPs. This approach holds promise for accelerating the in vivo delivery of large-sized pDNA and advancing the development of artificial cells.

## Introduction

Lipid-based drug delivery systems, such as lipid nanoparticles (LNPs) and liposomes, are important technologies in a variety of research fields and industrial sectors.^1−5^ LNPs offer many advantages for nucleic acid delivery, including high transfection efficiency, selective organ targeting, and minimal cytotoxicity. For these reasons, siRNA (Onpattro) or mRNA-loaded LNPs have been approved as RNA interference drugs and mRNA vaccines (Comirnaty and SpikeVax) for COVID-19.^5−8^ To date, practical applications have primarily involved LNPs carrying short RNA chains, typically under 5 kilobase pairs (kbp). For example, Onpattro and mRNA vaccines have employed RNA chains with lengths of 21 base pairs and approximately 4300 nucleotides, respectively. However, the delivery and transfection of larger-sized plasmid DNA (pDNA) or genomes remain a significant challenge. Typically, as the size of the pDNA increases, the transfection efficiency decreases, accompanied by increased cytotoxicity.^9^ Advancements in methods for delivering and transfecting large-sized pDNA and genomes hold the promise of generating precisely engineered and functionalized cells, as well as artificial cells.^10^

Various chemical, biological, and physical methods have been employed for pDNA transfection, including LNPs, transfection reagents, viruses, and electroporation.^11−13^ While each of these methods has its own advantages and disadvantages, both the LNP-based method and electroporation stand out for their simplicity and potential for high transfection efficiency. Notably, electroporation avoids the need for endosomal escape, as it allows pDNA to be directly delivered to the cytosol through the pores in the cell membrane. Søndergaard et al. reported that cotransfection of large-sized 15 kbp pDNA with smaller fragments ranging from 1.8 to 6.5 kbp improved transfection efficiency using electroporation.^14^ The transfection efficiency increased to 21.4%, representing a 6.8-fold improvement compared with no cotransfection. Electroporation is a powerful technique; however, it is associated with high cytotoxicity and presents challenges for targeted in vivo delivery.

**Figure 1:**
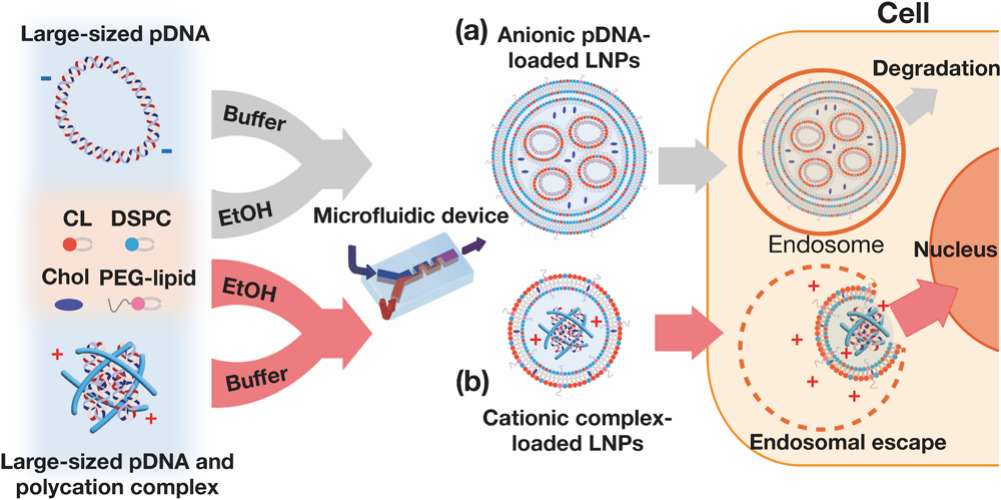
Schematic representation of large-sized pDNA transfection strategies employing (a) conventional LNPs and (b) polycation-pDNA complex-loaded LNPs. Both LNPs consist of ionizable (pH-sensitive cationic) or cationic lipid (CL), 1,2-distearoyl-*sn-glycero*-3-phosphocholine (DSPC), cholesterol (Chol), and 1,2-dimyristoyl-*rac*-glycero-3-methoxypolyethylene glycol-2000 (PEG-lipid). (a) Negatively charged pDNA was encapsulated in the LNPs. Large-sized pDNA could not be effectively released from the endosome into the cytosol. (b) LNPs loaded with positively charged pDNA-polycation core nanoparticles could accelerate endosomal escape.

LNP-based pDNA transfection involves several steps: cellular uptake, endosomal escape, nuclear translocation, transcription, and translation. In contrast, RNA transfection does not require nuclear translocation and transcription.^15^Figure 1a illustrates the conventional LNP-mediated pDNA transfection pathway. The negatively charged pDNA is encapsulated through the electrostatic interactions with a positively charged ionizable or cationic lipid.^16,17^ Following the cellular uptake of LNPs, pDNA is released from the endosome into the cytosol by membrane fusion or disruption. Generally, the process of endosomal escape is one of the most limiting steps in transfection. It is theorized that the efficiency of endosomal escape decreases as the size of pDNA increases due to its physical size. For small pDNA, even a minor fusion event between the lipid membrane and the endosomal membrane can lead to the release of pDNA from the endosome into the cytosol. In contrast, for large-sized pDNA, efficient release into the cytosol necessitates the dynamic fusion of LNPs with the endosomal membrane, which is more challenging. In addition, after successfully escaping the endosome, pDNA must pass through the nuclear membrane to reach the nucleus. In comparison with small-sized pDNA, large-sized pDNA has a lower diffusion rate in the cytosol and encounters greater difficulty in penetrating the nuclear membrane.^18^ While improved cellular uptake may enhance transfection efficiency, the critical factors involved in the transfection of large-sized pDNA are not well understood, and the efficient transfection of large-sized pDNA using LNPs has not yet been reported.

In this study, we present a method for transfecting large-sized pDNA using polymer–lipid hybrid nanoparticles. As illustrated in Figure 1b, we focused on compacting large-sized pDNA with polycations to improve the transfection efficiency. Unlike conventional compaction methods or hybrid nanoparticles, we have discovered that charge plays an important role in pDNA-polycation complexes. In the conventional method, negatively charged pDNA-polycation complexes are prepared and loaded into LNPs via electrostatic interactions with cationic lipids, which is considered the gold standard.^19,20^ This study aims to elucidate the effect of the charge of the pDNA-polycation complex on the transfection efficiency. Our findings demonstrate that positively charged complexes improve the transfection efficiency of large-sized pDNA. Additionally, through characterization and structure analysis of LNPs, we identified a small LNP size and a unilamellar structure as critical factors in facilitating the transfection of large-sized pDNA.

## Materials and Methods

### Materials

The pH-sensitive cationic lipid, CL15F6, was synthesized following the previously reported method.^21^ Cholesterol was purchased from Sigma-Aldrich (St. Louis, MO, USA). 1,2-Dioleoyl-3-trimethylammonium-propane (DOTAP), 1,2-di-*O*-octadecenyl-3-trimethylammonium propane (DOTMA), 1,2-dioleoyl-3-dimethylammonium-propane (DODAP), 1,2-dioleyloxy-3-dimethylaminopropane (DODMA), 1,2-distearoyl-*sn-glycero*-3-phosphocholine (DSPC), and 1,2-dimyristoyl-*rac*-glycero-3-methoxypolyethylene glycol-2000 (DMG-PEG 2k) were purchased from the NOF Corporation (Tokyo, Japan). DLin-MC3-DMA (MC3) was obtained from Selleck Biotech (Tokyo, Japan). Polyethylenimine (PEI), branched, molecular weight 10,000, was obtained from Thermo Fisher Scientific (Waltham, MA, USA). Ethanol, 2-morpholinoethanesulfonic acid (MES), monohydrate, D-PBS (−), sodium acetate, acetic acid, and sodium azide were purchased from FUJIFILM Wako Pure Chemical Corporation (Osaka, Japan). HeLa cells were obtained from the JCRB Cell Bank (Japan). DMEM and BSA were purchased from Sigma-Aldrich. Fetal bovine serum (FBS), BCA Protein Assay, penicillin-streptomycin, and trypsin (2.5%) were purchased from Thermo Fisher Scientific. CellTiter-Blue Cell Viability Assay, ONE-Glo Luciferase Assay System, Nano-Glo Luciferase Assay System, Glo Lysis Buffer, and the reporter plasmid pNL3.1[Nluc/minP] (3151 bp) encoding NanoLuc gene were purchased from Promega (Madison, WI, USA). pEF1a-2xSV40_NLS-NLuc was a gift from Antonio Amelio (Addgene plasmid #135953).^22^ HES7-NLuc-2A-tdTomato was a gift from James Thomson (Addgene plasmid #130932).^23^ pSLIK TT 3xFLAG Luciferase neo was a gift from Kevin Janes (Addgene plasmid #98392).^24^ pLV hU6-sgRNA hUbC-dCas9-KRAB-T2a-GFP was a gift from Charles Gersbach (Addgene plasmid #71237). Table 1 provides a summary of the pDNA sizes used in this study.^25^

**Table 1:** Summary of Model pDNAs Used in This Study

plasmid DNA	size (bp)	reporter protein
pNL3.1[Nluc/minP]	3151	nanoLuciferase
HES7-NLuc-2A-tdTomato	10,433	nanoLuciferase
pSLIK TT 3xFLAG luciferase neo	13,848	luciferase
pLV hU6-sgRNA hUbC-dCas9-KRAB-T2a-GFP	15,000	EGFP

### Preparation of LNPs

Lipid solutions were prepared by dissolving four types of lipids (either cationic lipid or ionizable lipid, DSPC, cholesterol, and DMG-PEG 2k) in ethanol. The total lipid concentration was 4 mM, with a composition of 60, 10, 30, and 1 mol % for cationic or ionizable lipid, DSPC, cholesterol, and DMG-PEG 2k, respectively. We used CL15F6, DOTAP, DOTMA, DODAP, DODMA, and MC3 as the cationic or ionizable lipids. For the aqueous phase, a 1 mg/mL PEI solution and 1 mg/mL pDNA solution was prepared using UltraPure DNase/RNase-Free Distilled Water (Thermo Fisher Scientific) and TE buffer. The pDNA solution was diluted to a concentration of 44 μg/mL with a 25 mM acetate buffer (pH 4.0). The PEI solution was diluted with acetate buffer to achieve concentrations of 0, 4.4, 44, and 220 μg/mL. The diluted pDNA solution was added drop by drop into each concentration of the PEI solution while vortexing. The final concentrations of pDNA and PEI were 22 and 0, 2.2, 22, and 110 μg/mL, respectively.

The LNP preparation method using the microfluidic device was described previously.^26−28^ We maintained a total flow rate of 500 μL/min, with a flow rate ratio (aqueous phase to lipid phase) of 6. The resulting LNP suspension was dialyzed for at least 2 h against a 20 mM MES buffer solution (pH 6.0), followed by overnight dialysis against D-PBS (−) (pH 7.4) using dialysis membrane tubing (12–14 kDa MW cutoffs, Repligen Corporation (Waltham, MA)). The size and Z-potential of LNPs and pDNA-PEI complex were measured using a Zeta-sizer Nano ZS ZEN3600 instrument (Malvern, UK).

### Characterization of LNP Structures

The internal structure of the LNPs was observed using transmission electron microscopy (TEM, HITACHI H-7600) at an acceleration energy of 100 kV. A suspension of LNPs was dropped onto a carbon-coated copper grid (400 mesh) and stained with a 2% phosphotungstic acid solution. TEM images were acquired by using a CCD camera (XR16, AMT imaging). Small-angle X-ray scattering (SAXS) measurements were performed on the beamline BL15A2 at the Photon Factory (Ibaraki, Japan).^29,30^ A suspension of LNPs was introduced into a flow cell, and X-rays were directed onto the flow cell to generate SAXS data. A wavelength of 1.2 Å was selected, and the X-ray detector (PILATUS 2M, DECTRIS, Switzerland) distance was set to 1.5 m. SAXS data were collected with a 1 s exposure time, integrated over 1200 images.

### In Vitro Assays

HeLa cells were cultured in DMEM supplemented with 10% FBS, 100 U/mL penicillin, and 100 μg/mL streptomycin (DMEM (+)) at 37 °C in a 5% CO_2_ incubator. To perform cell viability assays, HeLa cells were seeded into 96-well plates (4000 cells per well) and allowed to grow for 24 h. Prior to conducting the cell viability assay, LNP suspensions were diluted with DMEM (+) to a final concentration of 0.5 μg/mL. The cell culture medium was replaced with 100 μL of the prepared LNP suspension, followed by incubation for 24 h at 37 °C. After the transfection, the culture medium was replaced with 100 μL of fresh DMEM (+), and the cell viability was assessed using the CellTiter-Blue Cell Viability Assay Kit, following the manufacturer’s protocol.

To evaluate the transfection efficiency, HeLa cells were cultured in a 24-well plate (20,000 cells per well) 24 h before the experiments. The culture medium was then replaced with 1 mL of the LNP suspension, and the cells were incubated for 24 h at 37 °C. After the transfection, the culture medium was replaced with 500 μL of DMEM (+) and the cells were further incubated for 24 h. Subsequently, the culture medium was removed, and the HeLa cells were washed with PBS twice. For 15 kbp pDNA (EGFP encoded) transfection, GFP expression was assessed using flow cytometry (CytoFLEX Systems, Beckman Coulter, USA). The cells were detached with trypsin and then centrifuged (400*g*, 4 °C, 5 min), and the cell pellet was resuspended in 500 μL of FACS buffer, followed by filtration using a nylon mesh to remove debris. The acquired data were analyzed using Kaluza (Beckman Coulter). For other pDNA transfections, 100 μL of Glo Lysis Buffer was added to microwells following transfection and PBS washes. The lysate was then centrifuged at 24,660*g* and 4 °C for 2 min. Nluc or luciferase expression was assessed using the Nano-Glo Luciferase Assay System or ONE-Glo Luciferase Assay System, following the manufacturer’s protocols. The total protein content in the cell lysates was measured using a BCA Protein Assay, following the manufacturer’s protocol.

### Cellular Uptake Measurement by Confocal Laser Scanning Microscopy

HeLa cells expressing EGFP-EEA1 were treated with DiD-labeled LNPs encapsulating 15 kbp pDNA for 30 min at 37 °C and fixed with paraformaldehyde.^31^ Images were acquired with an IX83 microscope (Evident, Tokyo, Japan) equipped with a BioPoint MAC 6000 filter and shutter control unit (Ludl Electronic Products, Hawthorne, NY, USA), an automated XY-stage (Chuo Precision Industrial, Tokyo, Japan), a UPlanSApo 60×/1.35 oil objective lens, an X-lightV2 (CrestOptics, Rome, Italy) spinning-disk confocal unit, and a Zyla 4.2 scientific complementary metal-oxide semiconductor (sCMOS) camera (Oxford Instruments, Abingdon-on-Thames, UK). The cells were illuminated with an LDI laser light source (Chroma Technology Corp., Bellows Falls, VT, USA) through a ZET405/470/555/640x excitation filter (Chroma Technology Corp.). Emission filters were selected as follows: ET440/40m for Hoechst 33342, ET525/50m for EGFP, and ET700/75m for DiD-labeled LNPs. A ZT385/430/475/525/630 dichroic mirror (Chroma Technology Corp.) was used throughout imaging analysis. Control of the microscopes and peripheral equipment was managed by using MetaMorph software (Molecular Devices). Vesicle structures in DiD images (LNP) and EGFP images (EEA1) were extracted using the “Granularity” module of the MetaMorph software. The colocalization area for LNP and EEA1 was quantified using the “measure colocalization” function of the software.

## Results and Discussion

### Characterization of Large-Sized pDNA-Loaded LNPs

Table 1 provides an overview of the pDNA used in this study. A 15 kbp EGFP-coded plasmid was used as a model for large-sized pDNA.^14^ The process of endosomal escape poses the greatest challenge for large-sized pDNA and RNA transfection. As previously noted, the efficiency of endosomal escape is lower for large-sized pDNAs than for their smaller counterparts and RNAs. We investigated the transfection efficiency of LNPs loaded with 15 kbp pDNA using three types of ionizable lipids: CL15F6,^21^ SM-102,^32^ and D-Lin-MC3-DMA (MC3),^33^ as illustrated in Figure S1. CL15F6 was selected from our ionizable lipid library based on the hypothesis that a larger, hydrophobic alkyl tail group would be more effective for pDNA transfection. SM-102 and MC3 were selected as benchmark references for CL15F6. As expected, the transfection efficiency was low (below 10%) for all three LNPs. However, as we will detail in subsequent experimental results, we hypothesized that compacting large-sized pDNA with polycations, specifically branched polyethylenimine, could accelerate endosomal escape and thereby enhance transfection efficiency. In addition, we focused on evaluating the effect of the electric charge of the pDNA-PEI complex on transfection efficiency, as it led to changes in LNP characteristics including size and inner structure.

**Figure 2:**
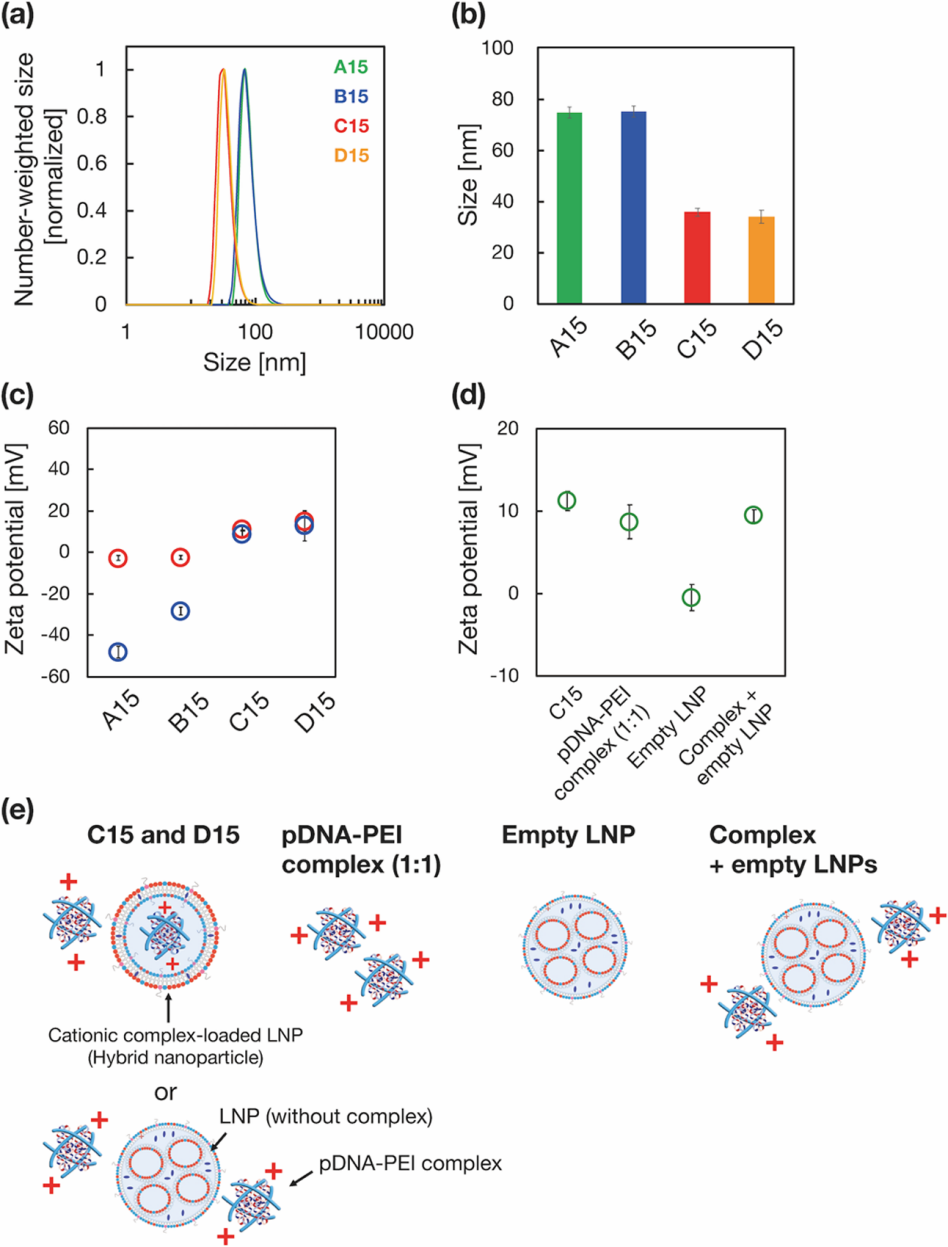
Characterization of 15 kb pDNA-PEI complex-loaded LNPs with CL15F6 as the ionizable lipid. (a) Size distributions and (b) average sizes of 15 kb pDNA-PEI complex-loaded LNPs. CL15F6 was used as an ionizable lipid. The mass ratios of pDNA to PEI were 1:0, 1:0.1, 1:1, and 5:1 for A15, B15, C15, and D15. (c) Zeta potentials of pDNA and pDNA-PEI complexes (blue circles) and pDNA (A15) or pDNA-PEI complex-loaded LNPs (B, C, and D-15, red circles) (*n* = 3). (d) Zeta potentials of C15, pDNA-PEI complex, empty LNP, and pDNA-PEI complex with empty LNP (*n* = 3). (e) Predicted states of C15 and D15 LNPs from the zeta potential. The zeta potential higher than 0 mV indicates that the pDNA-PEI complex was not completely loaded into the LNPs. Data are presented as the mean ± SD (*n* = 3).

Figure 2a,b presents the size distributions and average sizes of the four types of 15 kbp pDNA-loaded LNPs. The size of LNPs was measured by dynamic light scattering using the Zeta-sizer Nano ZS. These were formulated with different mixing ratios of pDNA to PEI, specifically 1:0, 1:0.1, 1:1, and 1:5 (w/w) for A15, B15, C15, and D15 LNPs, respectively. All LNPs had a monodisperse and narrow size distribution; however, the LNP size was influenced by the mixing ratio of pDNA and PEI. The size of the B15 LNP was 75 nm, similar to that of the A15 LNP, which was not complexed with PEI. In contrast, the C15 and D15 LNPs, which were complexed with high amounts of PEI, measured 35 nm in size. Figure 2 presents the zeta potential of the pDNA-PEI complex before (blue) and after (red) encapsulation into LNPs. Naked pDNA and the complex in a 1:0.1 ratio (conditions A and B) had a negative charge ranging from −30 to −50 mV. These pDNA and PEI complexes effectively undergo encapsulation into LNPs through electrostatic interactions between the negatively charged complex and the ionizable lipid, CL15F6. Following encapsulation into LNPs (A15 and B15), the zeta potentials increased to almost 0 mV. In contrast, C15 and D15 LNPs and their complexes had positive charges of 10 mV. To further investigate the characteristics of the C15 and D15 LNPs, the zeta potentials of the complex, LNP without the complex (empty LNP), and the mixture of the complex and empty LNP were measured. Figure 2 presents the zeta potentials of the particles and the proposed states of the C15 and D15 LNPs based on the zeta potential data. The zeta potentials of the pDNA-PEI complex and the mixture of the complex and empty LNP were the same as that of the C15 LNP. In contrast, the empty LNP had a zeta potential of 0 mV. These results suggest that LNPs formulated with the ionizable lipid CL15F6 might not entirely encapsulate the cationic pDNA-PEI complex. We hypothesized that the electric charge of the core particles affects the size, internal structure, and transfection performance of LNPs.

### Cellular Uptake Analysis

**Figure 3:**
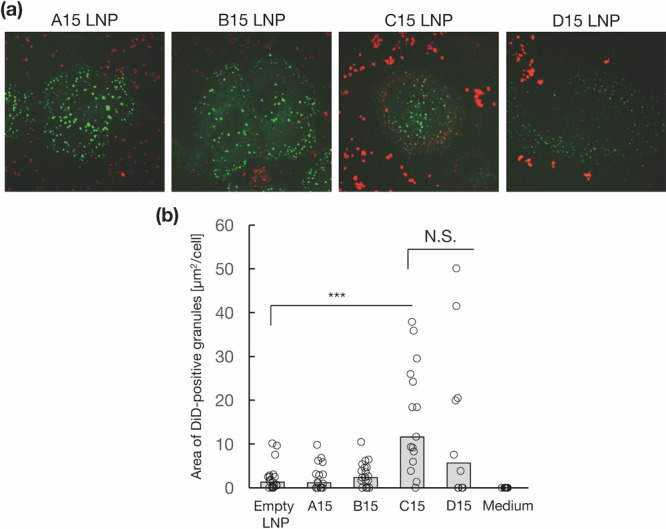
(a) Representative images and (b) quantification of the cellular uptake of DiD-labeled LNPs. LNPs are shown in red, while the endosome marker EGFP-EEA1 is green. Data are presented as the mean ± SD (*n* = 3). ***: *P* < 0.001. N.S.: not significant.

The size of the LNPs plays an important role in cellular uptake efficiency; therefore, we evaluated the cellular uptake of each LNP type. Figure 3a presents the cellular uptake of DiD-labeled LNPs (red), and colocalization with the endosome marker EEA1 (EGFP, green).^34^ Compared with other LNPs, C15 LNPs were clearly colocalized with endosomes. Based on quantification of the colocalized area between LNPs and EEA1, both C15 and D15 LNPs demonstrated significantly higher cellular uptake compared with A15 and B15 LNPs (Figure 3b). Existing literature suggests that smaller nanoparticles are internalized more easily than their larger counterparts.^35^ In our experiments, A-B15 LNPs were 75 nm in size, while C–D 15 LNPs were 35 nm. Consequently, the 35 nm LNPs (C and D15) promoted greater cellular uptake and colocalization with endosome markers. These findings highlight the significance of LNP size in improving the transfection efficiency of large-size pDNA.

### Transfection of Large-Sized pDNA

**Figure 4:**
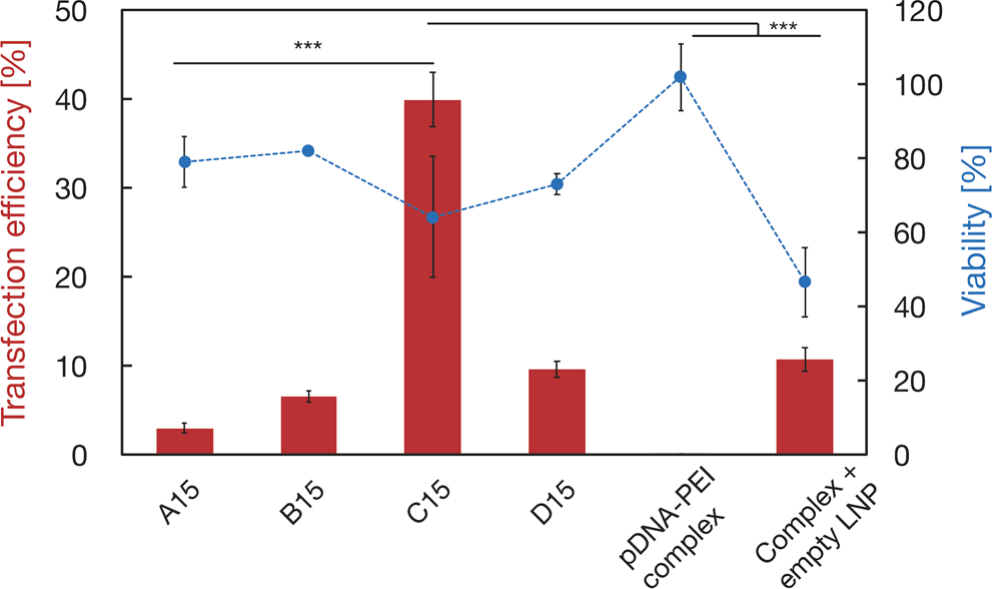
Transfection efficiencies (bar chart) and cell viability (plots) of each LNP. Data are presented as the mean ± SD (*n* = 3). ***: *P* < 0.001 (for transfection efficiency).

The transfection efficiency of the nanoparticles loaded with 15 kbp pDNA (EGFP encoded) and associated cell viability was assessed for the different LNPs (Figure 4). The C15 LNP (pDNA:PEI = 1:1) achieved a 40% transfection efficiency, which was eight times greater than that of the conventional A15 LNP (pDNA:PEI = 1:0). Furthermore, the cell viability for the C15 LNP was comparable to that of the A15 LNP. In contrast, the transfection efficiencies of both the B15 LNP (pDNA:PEI = 1:0.1) and D15 LNP (pDNA:PEI = 1:5) were comparable to that of the A15 LNP. To investigate the impact of the pDNA to PEI mixing ratio, nine types of LNPs were prepared with mixing ratios ranging from 1:0 to 1:10. Interestingly, the 1:1 mixing ratio (C15 LNP) resulted in a high transfection efficiency (Figure S2). We also evaluated the transfection performance of the pDNA-PEI complex (without LNPs) and the complex with empty LNPs to elucidate the characteristics of the C15 and D15 LNPs (Figure 2e). The pDNA-PEI complex did not express EGFP, although the transfection efficiency slightly increased with the addition of empty LNPs, reaching the same level as that of the D15-LNP. The viability of cells treated with the pDNA-PEI complex was almost 100%, indicating limited cellular uptake of the pDNA-PEI complex. These results strongly suggest that the C15 LNPs encapsulate cationic-charged pDNA-PEI complexes, coexisting with free pDNA-PEI complexes in the suspension (Figure 2e upper-left).

**Figure 5:**
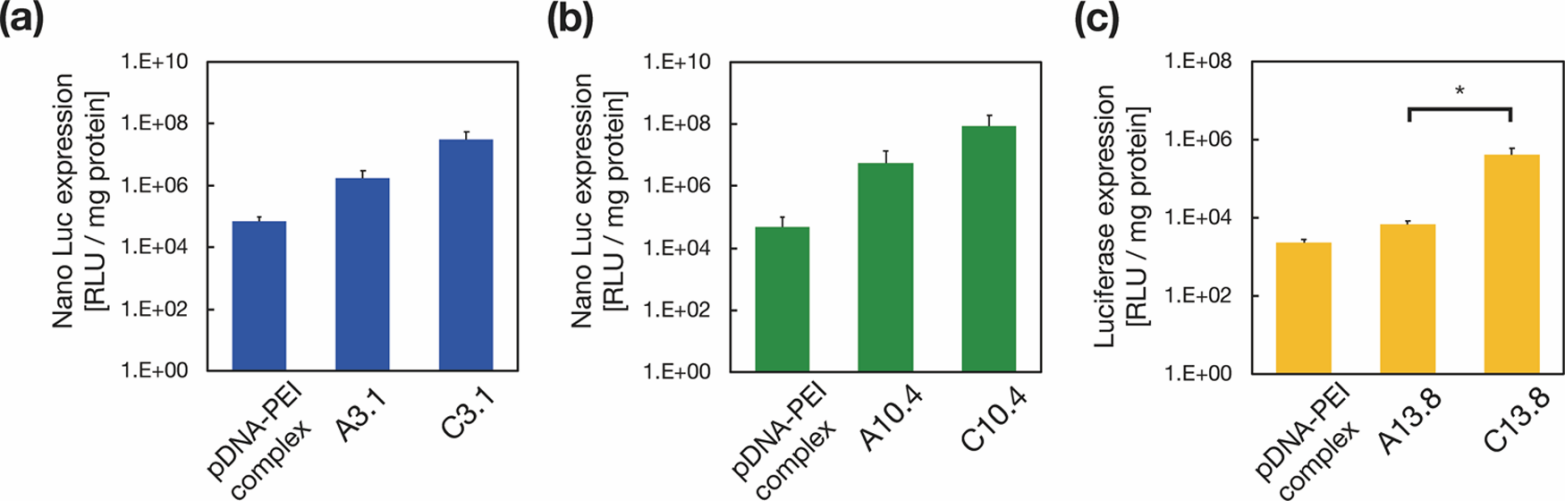
Comparison of transfection efficiencies using three types of particles: pDNA-PEI complex, pDNA-loaded LNPs (A-LNP), and LNPs loaded with pDNA-PEI complex (1:1, C-LNP) using (a) 3.1 kbp (blue), (b) 10.4 kbp (green), and (c) 13.8 kbp (yellow) pDNAs. Data are presented as the mean ± SD (*n* = 3). *: *P* < 0.05. N.S.: not significant.

Our findings indicate that LNPs exhibit high transfection efficiency only when branched PEI was used as a polycation. Other polycations such as stearylated R8,^36^ protamine sulfate,^37^ and linear polyethylenimine^38^ did not enhance the transfection efficiency (Figure S3). We also compared the transfection efficiency of four types of particles: pDNA-PEI complex, pDNA-loaded LNPs (A-LNP), and LNPs loaded with pDNA-PEI complex (1:1, C-LNP) using 3.1, 10.4, and 13.8 kbp pDNA. The pDNA-PEI complex had a low transfection efficiency for any size of pDNAs (Figure 5). In contrast, A- and C-LNPs demonstrated higher transfection performance than the pDNA-PEI complex. Interestingly, when comparing A-LNP and C-LNP, the transfection efficiency of C-LNPs increased with increasing pDNA size. The average transfection efficiencies of C-LNPs were 6, 16, and 58-fold higher than those of the A-LNPs, for 3.1, 10.4, and 13.8 kbp pDNAs, respectively. Typically, negatively charged pDNA or pDNA-polycation complexes are mixed with a lipid solution containing an ionizable lipid as the main component, and encapsulate into LNPs through electrostatic interactions.^19,20^ However, we have established that this approach cannot effectively transfect large-size pDNA and determined that the encapsulated structure of the cationic-charged pDNA-PEI complex plays an essential role in large pDNA transfection.

To elucidate the key factors responsible for the high transfection efficiency, a hemolysis assay was performed and the effect of free PEI on transfection efficiency was evaluated (Figures S4 and S5). At pH levels ranging from 5.0 to 6.0, C15 and D15 LNPs exhibited greater hemolytic activity compared to A15 and B15 LNPs. Notably, the pDNA-PEI complexes did not show hemolytic activity. These results suggest that C15 and D15 LNPs had a higher percentage of CL15F6 on their surfaces, potentially facilitating endosomal escape. According to the literature, free PEI coexists in the pDNA-PEI solution and enhances the transfection efficiency.^38,39^ As shown in Figure S5, the cotransfection of free PEI or the pDNA-PEI complex (panels a and b) with A15 LNP increased the transfection efficiency from 3 to 10%; however, the transfection efficiency did not reach the level achieved with C15-LNP (40%). Based on these results, we successfully established a platform for transfecting large pDNA using polymer–lipid hybrid nanoparticles. To gain a better understanding of how these hybrid nanoparticles impact the transfection of large-sized pDNA, we investigated the inner structures of the hybrid nanoparticles.

### Characterization of the Internal Structure of Large-Sized pDNA-Loaded LNPs

Based on measurements of LNP size and zeta potential, we categorized A15 and B15 as large-sized LNPs, while C15 and D15 were classified as small-sized LNPs. A15 (without PEI) and B15 (pDNA:PEI = 1:0.1) encapsulated negatively charged pDNA or pDNA-PEI complexes into the nanoparticles. Conversely, C15 and D15 encapsulated a positively charged pDNA-PEI complex into the nanoparticles. Transfection experiments revealed that the C15 LNP has more favorable characteristics for transfecting large-size pDNA. Therefore, we expected differences in the internal structures of A-B15 and C-D15 due to electrostatic interactions or repulsion. Typically, LNPs encapsulating negatively charged pDNA and RNAs, such as A15 LNP, have multilamellar structures formed through the interactions between ionizable lipids and pDNA or RNA.^40,41^

**Figure 6:**
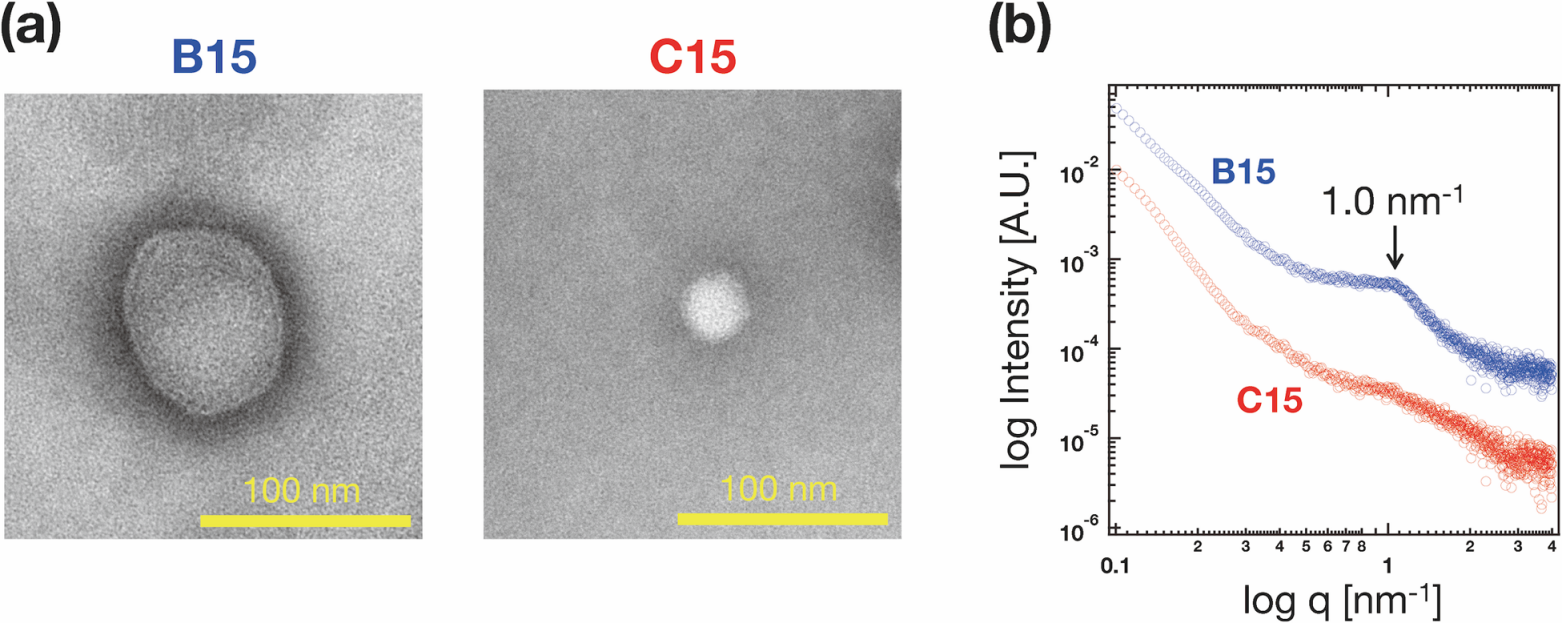
(a) Transmission microscopy (TEM) images and (b) small-angle X-ray scattering (SAXS) analysis of the B15 and C15 LNPs. Each scale bar represents 100 nm.

The internal structures of B15 and C15 LNPs were analyzed using transmission microscopy (TEM) and small-angle X-ray scattering (SAXS) analysis (Figure 6). TEM analysis confirmed that the sizes of the B15 and C15 LNPs were consistent with those obtained from dynamic light scattering (DLS) measurements, as shown in Figure 2a. However, the internal structures of B15 and C15 LNPs differed significantly. The internal structures of B15 and C15 LNPs exhibited a combination of multilamellar and unilamellar liposome-like structures. Figure 6b presents the SAXS profiles of the B15 and C15 LNPs. The SAXS peak at 1.0 nm^–1^ corresponds to the ordered lamellar structure (*d*-spacing of 6.3 nm), observed in B15 LNPs.^29^ In contrast, the C15 LNP did not exhibit specific SAXS peaks associated with structural features. These results highlight the influence of the pDNA/PEI mixing ratio on both the size and internal structure of LNPs. Furthermore, they suggest that a small particle size and unilamellar structure are the optimal physical properties for transfecting large-size pDNA.

### Impact of Ionizable and Cationic Lipids on the Transfection of Large-Sized pDNA

**Figure 7:**
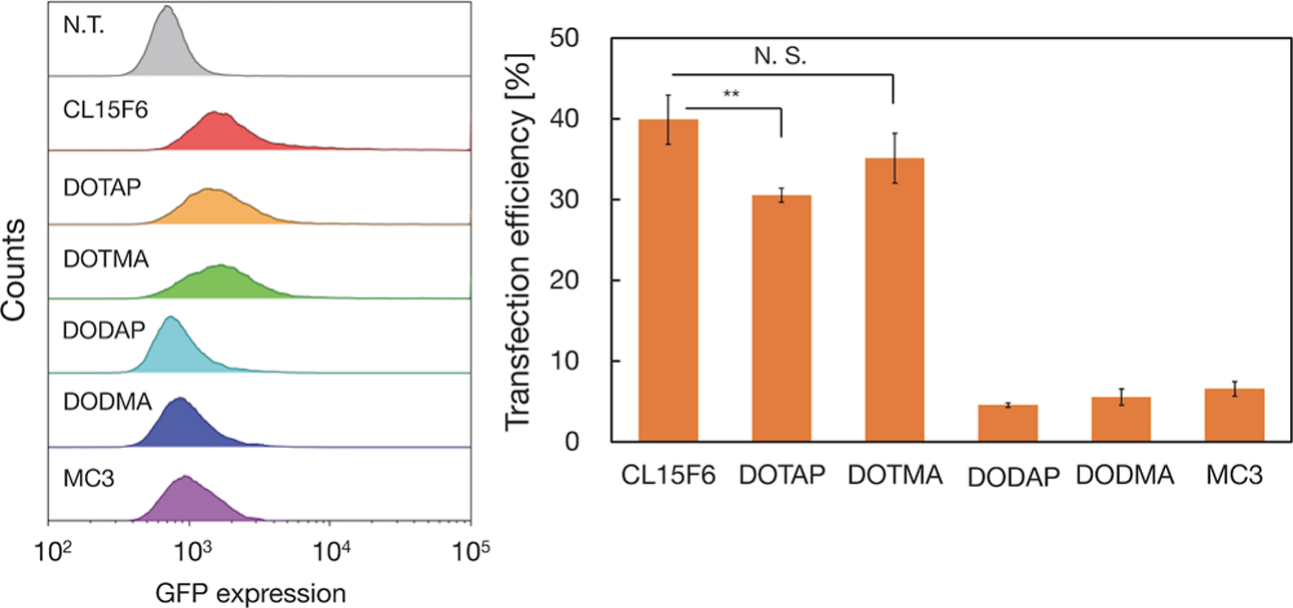
Comparison of the transfection efficiency of the six types of LNP–polymer hybrid nanoparticles. (left) GFP expression determined by flow cytometry. (right) Average transfection efficiencies of each LNP. Ionizable lipids: CL15F6, DODAP, DODMA, and MC3; cationic lipids: DOTAP and DOTMA. N.T.: nontreated. Data are presented as the mean ± SD (*n* = 3). **: *P* < 0.005.

The hemolysis assay and transfection performance analysis with A15 LNP, C15 LNP, and pDNA-PEI complexes (Figures S4 and S5) indicated that the presence of ionizable lipids at the LNP surface plays an essential role in facilitating endosomal escape for large-sized pDNA transfection. To further explore the influence of different types of ionizable and cationic lipids on large-sized pDNA transfection, we examined commercially available cationic lipids (DOTAP and DOTMA) and ionizable lipids (DODAP, DODMA, and MC3) (Figure S6). The nanoparticle size was approximately 40 nm, similar to that of the CL15F6-based nanoparticles. The zeta potentials of the DOTAP- and DOTMA-based nanoparticles were +20 mV, exceeding those of the ionizable lipid-based nanoparticles (Figure S7). Figure 7 presents a comparative analysis of transfection efficiency among the six types of LNP–polymer hybrid nanoparticles. The transfection efficiency of CL15F6, DOTAP, and DOTMA-based nanoparticles was approximately 40%, whereas the transfection efficiency of other ionized lipid-based nanoparticles was less than 10%. This result differs significantly from that of transfection with short nucleic acids such as siRNA. The ionizable lipid MC3 was developed to maximize gene knockdown performance by siRNA and was approved as the first RNA interference drug. However, its transfection performance for large-sized pDNA was comparable to that of other ionizable lipids (DODAP and DODMA), and inferior to that of cationic lipid-based nanoparticles. Among the ionizable lipids, only CL15F6 exhibited a high transfection efficiency. We posit that the bulky alkyl group in CL15F6 induced dynamic endosomal membrane disruption, thereby enhancing the endosomal escape of the large pDNA. Furthermore, we identified the nanoparticle surface charge as a critical factor in improving the transfection efficiency of large-sized pDNA. The cationic charge of the nanoparticles also enhances the disruption of the endosomal membrane.

### Transfection Mechanism of Large-Sized pDNA Using Lipid–Polymer Hybrid Nanoparticles

**Figure 8:**
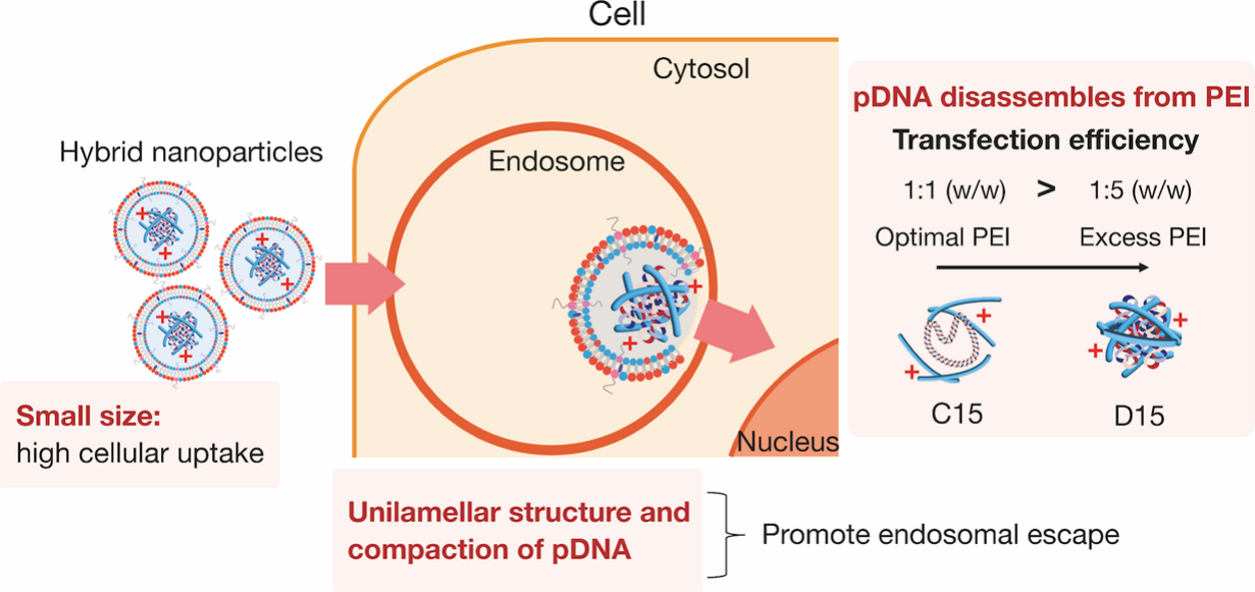
Proposed mechanism illustrating the effective transfection of large-sized pDNA facilitated by lipid–polymer hybrid nanoparticles. Compaction of pDNA with PEI coupled with a unilamellar structure and a cationic nanoparticle charge can accelerate the endosomal escape of large-sized pDNA.

In this study, we identified three key factors for the effective transfection of large-sized pDNA. Figure 8 illustrates the hypothetical mechanism underlying the transfection of large-sized pDNA using lipid–polymer hybrid nanoparticles. First, cells efficiently internalize small-sized nanoparticles. Second, the unilamellar structure and compaction of large-sized pDNA by PEI facilitate endosomal escape. We suggest that dynamic fusion or disruption of the endosomal membrane is required for the effective release of large-sized pDNA into the cytosol, especially when compared with the small-sized RNA and pDNA. The compaction of pDNA with PEI reduces its physical size, facilitating its release into the cytosol with minimal disruption of the endosomal membranes. In addition, the unilamellar structure proves more advantageous for the release of large-sized pDNA into the cytosol when compared to a multilamellar structure. In a multilayered structure, the pDNA-PEI complex must pass through a number of LNP membranes before it can be released into the cytoplasm. Furthermore, within these multilayers, electrostatic interactions between negatively charged pDNA or pDNA-PEI complexes and ionized lipids may prevent their release into the cytoplasm. Therefore, the compaction of pDNA with PEI, coupled with a unilamellar structure and a cationic nanoparticle charge, can accelerate the endosomal escape of large-sized pDNA. These factors hold greater importance in the delivery of large-sized pDNA compared to small-sized pDNA or RNAs. The third factor involves the disassembly of pDNA from PEI. Once the pDNA-PEI complex is transported into the nucleus, the pDNA needs to be separated from the PEI for transcription to occur. Therefore, pDNA cannot dissociate from the complex under conditions of excess PEI, as observed in D15 LNPs (pDNA:PEI = 1:5). This assumption is further supported by the experimental results (Figure S2). The transfection efficiency was maximized at a pDNA to PEI ratio of 1:1 and decreased with an increased PEI concentration. We propose that the combined and synergistic effects of these three factors contribute to the high transfection performance of large-sized pDNA using polymer–lipid hybrid nanoparticles.

## Conclusions

In this study, we developed polymer–lipid hybrid nanoparticles to facilitate the transfection of large-sized pDNA. In addition, we identified the key factors that enhance transfection efficiency and proposed a possible mechanism for large-sized pDNA transfection. Conventional LNP-based strategies, which rely on electrostatic interactions to encapsulate negatively charged pDNA or pDNA-PEI complexes using cationic or ionizable lipids, do not achieve a high transfection performance. We determined that LNPs encapsulating a cationic pDNA-PEI core at an optimal pDNA to PEI ratio promoted the high transfection efficiency of large-sized pDNA. These polymer–lipid hybrid nanoparticles exhibited distinct characteristics, notably a small size and unilamellar structure, distinguishing them from LNPs prepared by conventional methods. Moreover, among the four types of ionizable lipids tested, only the CL15F6-based hybrid nanoparticles demonstrated an improvement in the transfection performance. The bulky alkyl group structure may facilitate the endosomal escape of large-sized pDNA. Further studies are required to obtain a detailed understanding of the transfection mechanism, including intercellular observations, assessment of transcription efficiency, and in vivo studies. However, our findings propose a new delivery or transfection strategy for large-sized pDNA, mRNA, or genome that is distinct from conventional LNP-based approaches. We anticipate that the development of new polycations and optimized ionizable or cationic lipids for large-sized pDNA delivery, combined with our hybrid nanoparticle strategy, will further advance the field of large-sized DNA or genome delivery, benefiting various research communities and applications.

### Statistical Analysis

Experimental results were analyzed using Microsoft Excel. Statistical significance was defined as *P* values <0.05. Data are presented as mean ± SD for the indicated number of experiments. Pair-wise comparisons between treatments were made using a two-tail Student's *t* test. For multiple comparisons, we employed ANOVA, followed by a Bonferroni test.

## Supporting Information Available

Results of conventional LNP-based transfection, impact of the mixing ratio of pDNA and PEI, effect of types of polycation on transfection efficiency, hemolysis assay, effect of free PEI and pDNA-PEI complex, molecular structure of ionizable and cationic lipids, and characteristics and transfection efficiency of six types of lipid-polymer hybrid nanoparticles (PDF)
