# Supplementary material for: Development of Polymer–Lipid Hybrid Nanoparticles for Large-Sized Plasmid DNA Transfection
Source: ACS Appl Mater Interfaces. 2023 Dec 23;16(2):2110–9. doi: 10.1021/acsami.3c14714 (PMC10798250; doi:10.1021/acsami.3c14714)
Supplement: Supplementary file 1 — am3c14714_si_001.pdf [file am3c14714_si_001.pdf]

Supporting Information

The Development of Polymer-Lipid Hybrid Nanoparticles for Large-Sized Plasmid DNA Transfection

Masatoshi Maeki,[a,b,c#] Shuya Uno,[d#] Kaisei Sugiura,[d] Yusuke Sato,[e] Yoichiro Fujioka,[f] Akihiko Ishida,[a] Yusuke Ohba,[f] Hideyoshi Harashima,[d] and Manabu Tokeshi[b*]*

[a] *Division of Applied Chemistry, Faculty of Engineering, Hokkaido University, Kita 13 Nishi 8, Kita-ku, Sapporo 060-8628, Japan*

[b] *JST PRESTO, 4-1-8 Honcho, Kawaguchi, Saitama 332-0012, Japan*

[c] *Institute of Materials Structure Science, High Energy Accelerator Research Organization (KEK), Tsukuba, Ibaraki 305-0801, Japan*

[d] *Graduate School of Chemical Sciences and Engineering, Hokkaido University, Kita 13 Nishi 8, Kita-ku, Sapporo 060-8628, Japan*

[e] *Faculty of Pharmaceutical Sciences, Hokkaido University, Kita 12 Nishi 8, Kita-ku, Sapporo 060-0812, Japan*

[f] *Department of Cell Physiology, Faculty of Medicine and Graduate School of Medicine, Hokkaido University, Kita 15 Nishi 7, Kita-ku, Sapporo 060-8638, Japan*

[#] *M.M. and S.U. contributed equally to this work.*

*Corresponding authors:

Masatoshi Maeki

E-mail address: m.maeki@eng.hokudai.ac.jp

Tel: +81-11-706-6773 Fax: +81-11-706-6745

Manabu Tokeshi

E-mail address: tokeshi@eng.hokudai.ac.jp

Tel: +81-11-706-6744 Fax: +81-11-706-6745

Conventional LNP-based Transfection

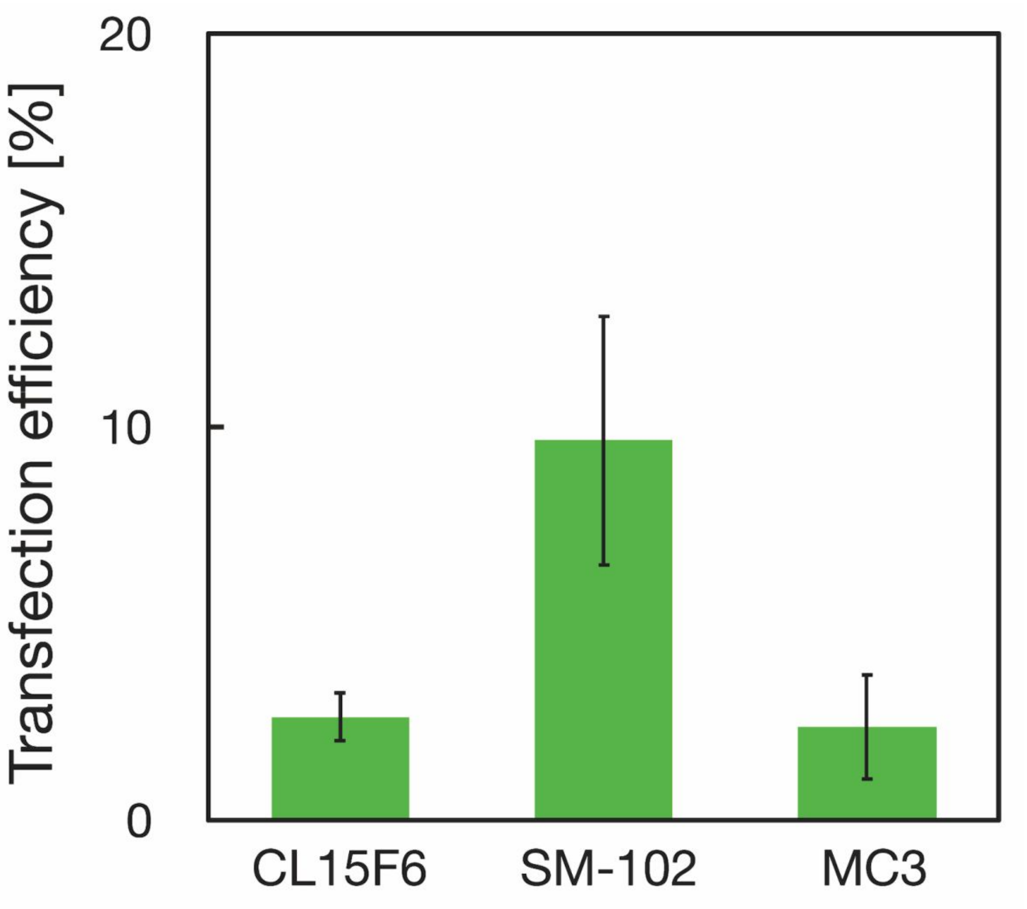

Figure S1. Transfection efficiency of 15 kbp-pDNA-loaded LNPs using three types of ionizable lipids, CL15F6, SM-102, and D-Lin-MC3-DMA (MC3). Data are presented as the mean ± SD (n = 3).

Impact of the Mixing Ratio of pDNA and PEI

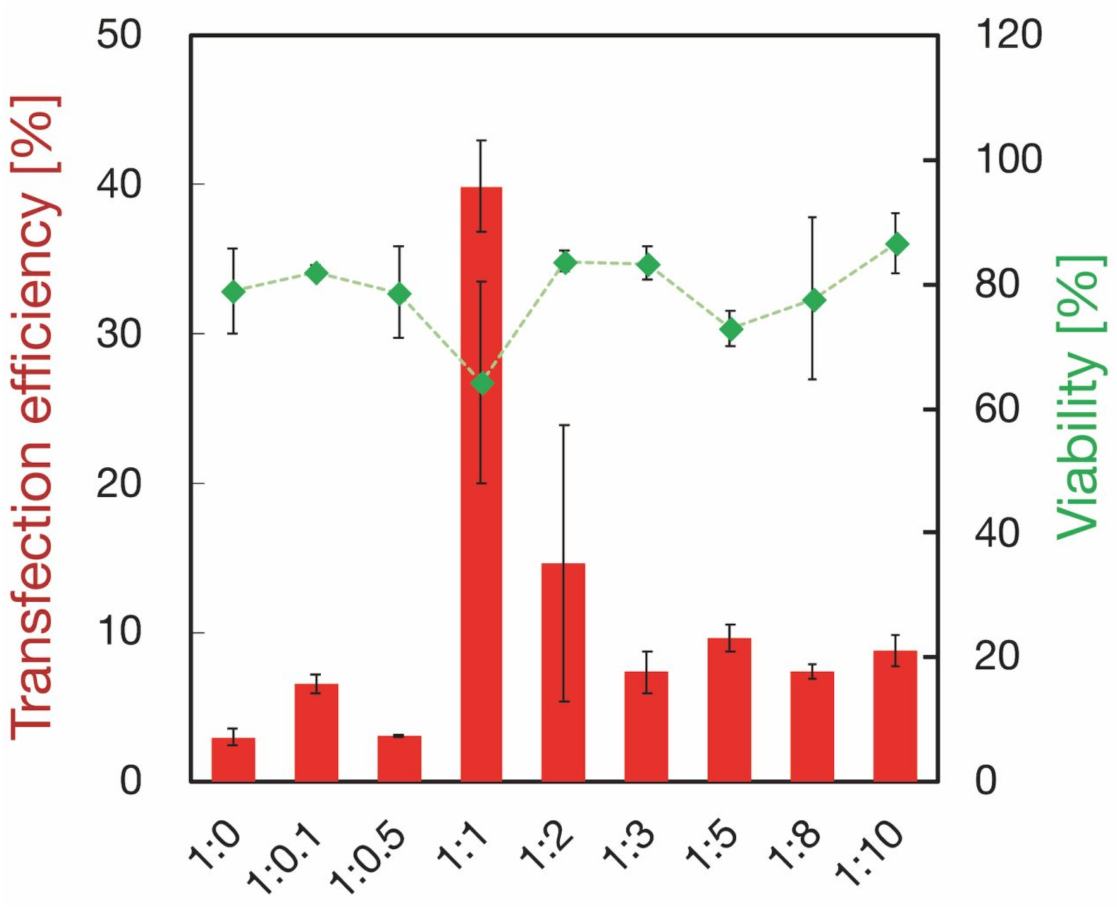

Figure S2. Impact of the mixing ratio of pDNA and PEI on the 15 kbp pDNA transfection efficiency (red) and cell viability (green). Mixing ratios were 1:0 to 1:10 (pDNA:PEI). Data are presented as the mean ± SD (n = 3).

Effect of Types of Polycation on Transfection Efficiency

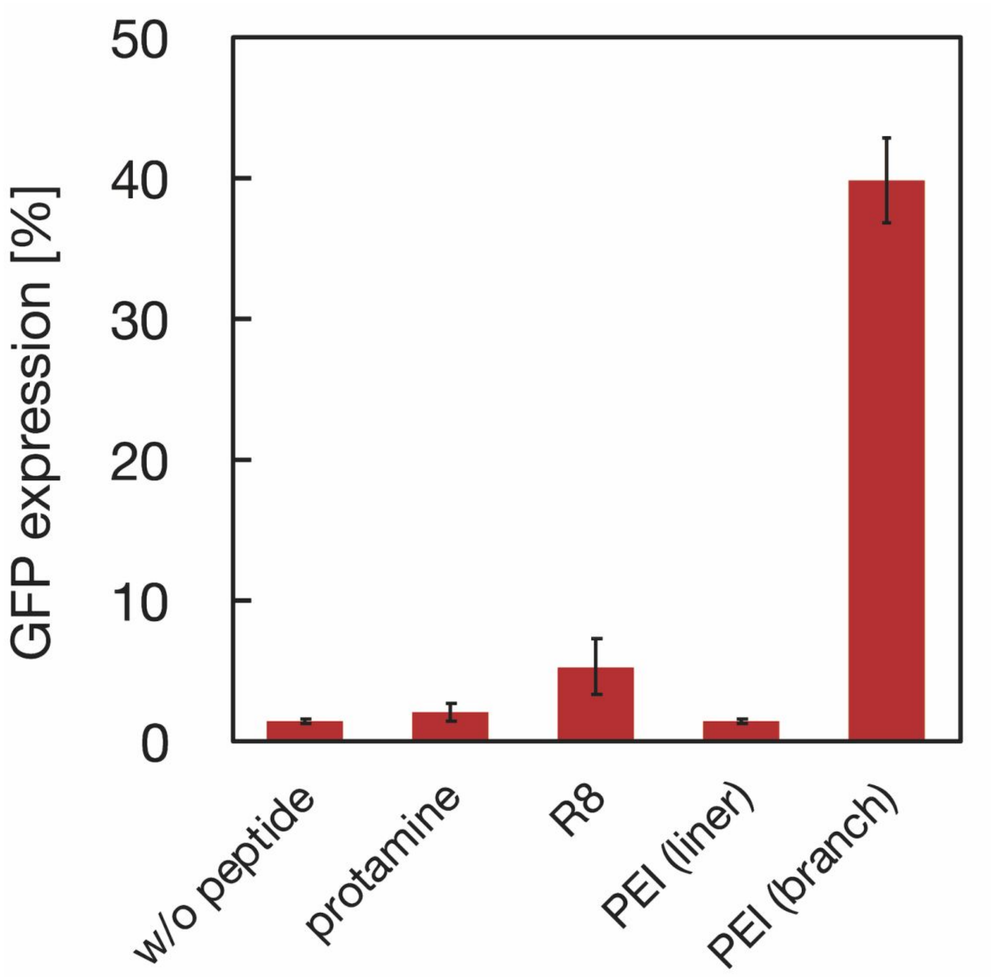

Figure S3. Comparison of 15 kbp pDNA transfection efficiencies using a variety of polycations. We observed that LNPs exhibit high transfection efficiency only when branched PEI was used as a polycation. Other polycations such as stearylated R8, protamine sulfate, and linear polyethyleneimine could not enhance the transfection efficiency. Data are presented as the mean ± SD (n = 3).

Hemolysis Assay

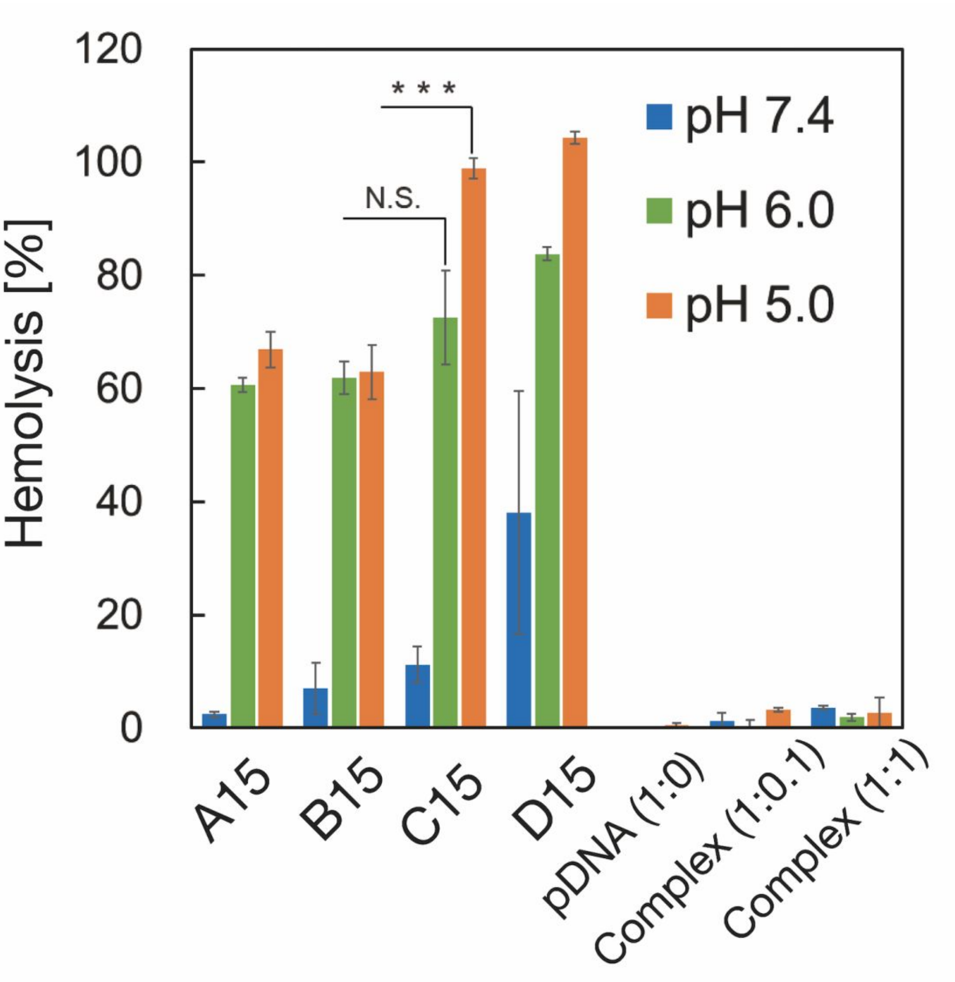

Figure S4. Hemolytic activities of A-D15 LNPs and pDNA-PEI complexes. C15 and D15 LNPs showed higher hemolytic activity than those of the A15 and B15-LNPs at the acidic condition, such as late endosome. Data are presented as the mean ± SD (n = 3). ***: $P < 0.001$. *N.S.*: not significant.

Effect of Free PEI and pDNA-PEI Complex

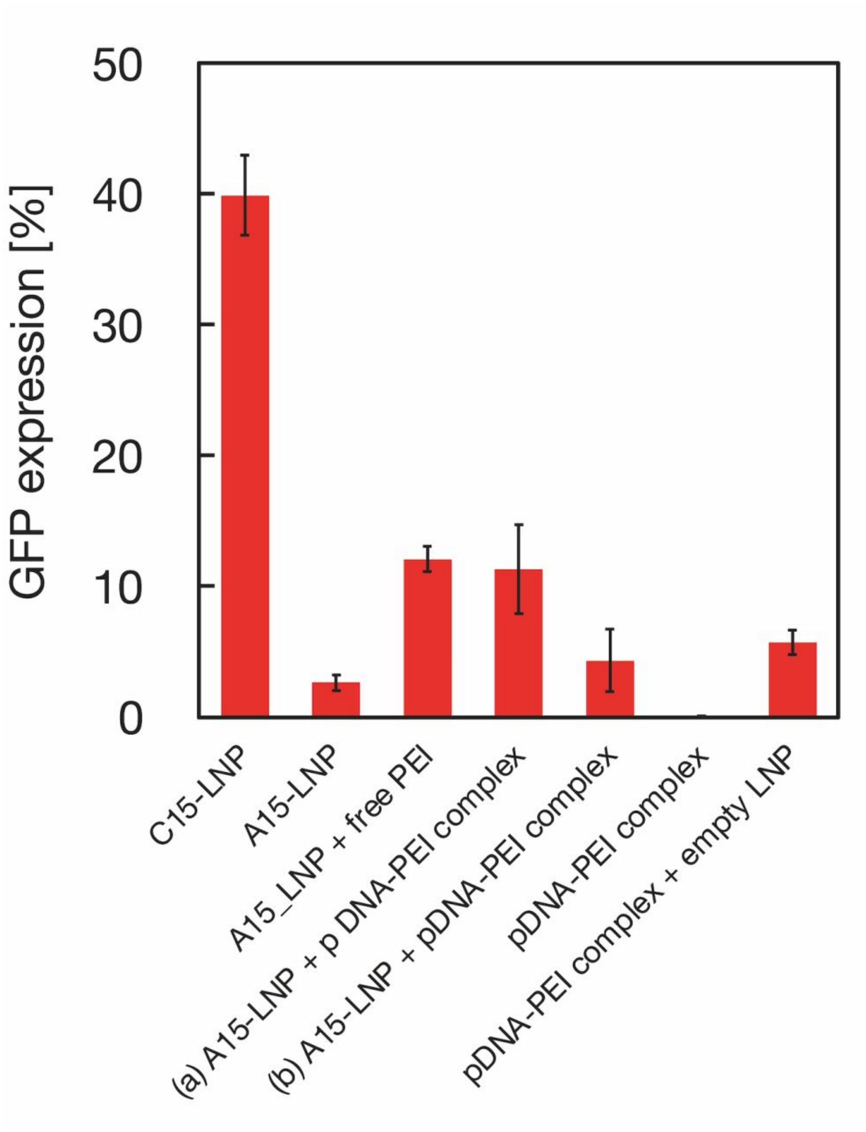

Figure S5 Effect of free PEI and pDNA-PEI complex on 15 kbp pDNA transfection. (a) Final concentration of pDNA was 1 μg/mL containing of 0.5 μg/mL A-15 LNP and 0.5 μg/mL pDNA-PEI complex. (b) Final concentration of pDNA was 0.5 μg/mL containing of 0.25 μg/mL A-15 LNP and 0.25 μg/mL pDNA-PEI complex. pDNA dose amount was 0.5 μg per well except for sample (a) A15-LNP + pDNA-PEI complex. Data are presented as the mean ± SD (n = 3).

Molecular Structure of Ionizable and Cationic Lipids

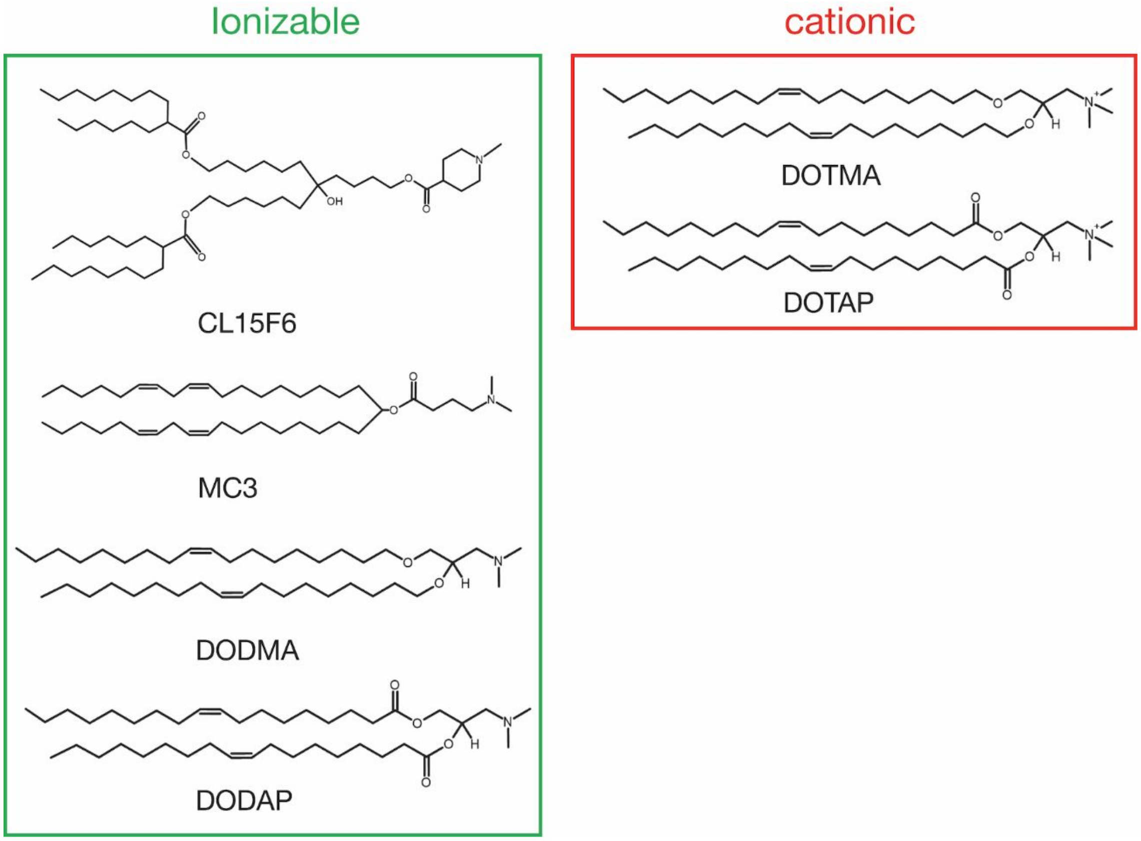

Figure S6. Ionizable and cationic lipids used in this study. Ionizable lipids: CL15F6, MC3, DODMA, and DODAP. Cationic lipids: DOTMA and DOTAP.

Characteristics and Transfection Efficiency of Six Types of Lipid-Polymer Hybrid Nanoparticles

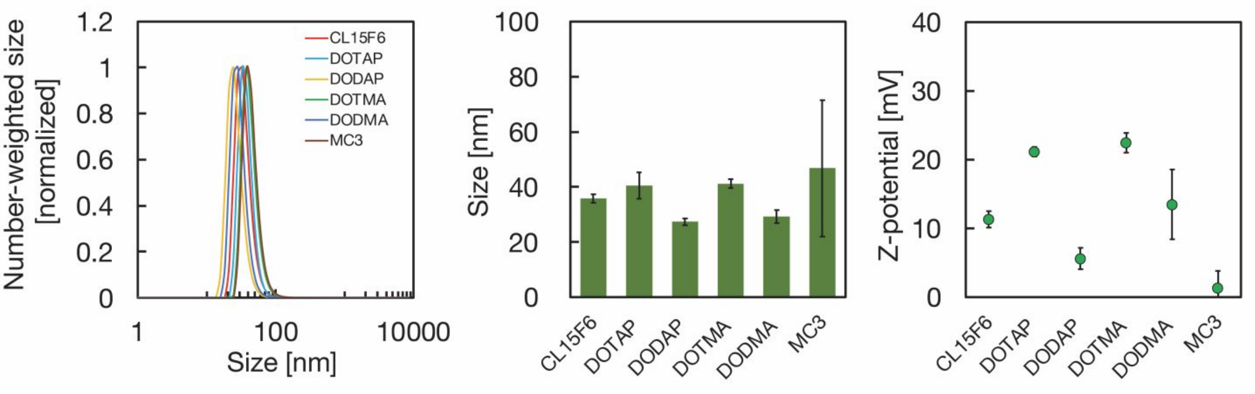

Figure S7. LNP size distributions, average-sizes, and Zeta potential of six types of lipid-polymer hybrid nanoparticles. Data are presented as the mean ± SD (n = 3).

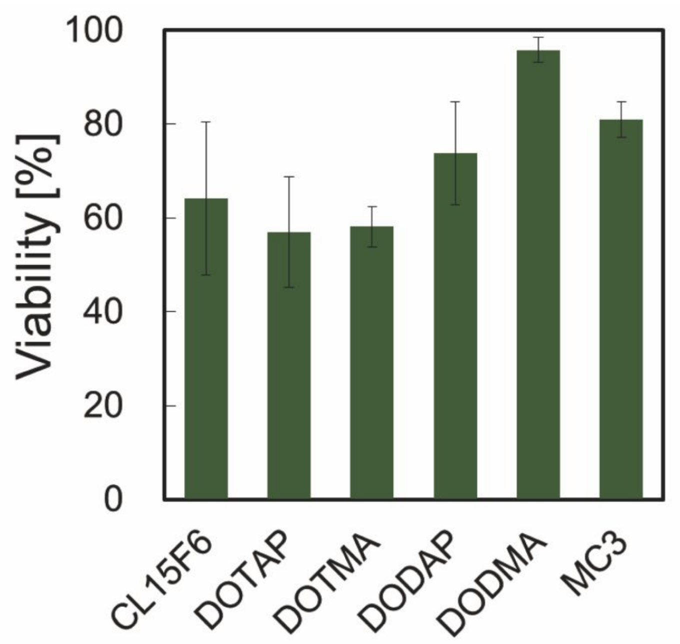

Figure S8. Transfection efficiencies and cell viability of 15 kbp pDNA-loaded six types of lipid-polymer hybrid nanoparticles. The data are represented as the mean ± SD (n = 3). **: $P < 0.005$. *N.S.*: not significant.
